# Mozart for the brain - a pilot study on physiological effects of auditive stimulation in patients after aneurysmal subarachnoid hemorrhage^☆^

**DOI:** 10.1016/j.ibneur.2025.06.008

**Published:** 2025-06-21

**Authors:** Nicolas Eden, Marius Marc-Daniel Mader, Jan Bremer, Jennifer Sauvigny, Jörn Grensemann, Marlene Fischer, Nils Schweingruber, Jens Gempt, Patrick Czorlich

**Affiliations:** a Department of Neurosurgery, University Medical Center Hamburg-Eppendorf, Martinistrasse 52, Hamburg 20246, Germany; b Department of Psychiatry and Psychotherapy, University Medical Center Hamburg-Eppendorf, Martinistrasse 52, Hamburg 20246, Germany; c Institute for Stem Cell Biology and Regenerative Medicine, Stanford University School of Medicine, Stanford, California, United States; d Department of Intensive Care Medicine, University Medical Center Hamburg-Eppendorf, Martinistrasse 52, Hamburg 20246, Germany; e Department of Neurology, University Medical Center Hamburg-Eppendorf, Martinistrasse 52, Hamburg 20246, Germany

**Keywords:** subarachnoid hemorrhage, physiological effects, vasospasm, classical music, Mozart

## Abstract

**Background:**

Classical music influences human physiology, such as the cerebral blood flow velocity (CBFV), in healthy controls and during recovery from ischemic stroke. Aim of this prospective pilot-study was to investigate the effect of classical music on CBFV and other physiological parameters in patients suffering from aneurysmal subarachnoid hemorrhage (SAH).

**Methods:**

Twenty patients with SAH were subjected to up to three interventions, in which the patients listened to *W. A. Mozart's Symphony No. 40 in G minor*. In parallel, CBFV in the right middle cerebral artery (MCA) was continuously measured using transcranial Doppler (TCD). TCD values were averaged per minute, normalized, and analyzed with a mixed-effects linear regression model. In addition, other physiological and laboratory parameters were evaluated.

**Results:**

A total of 55 interventions were successfully carried out. The mixed-effects linear regression model revealed significant associations with both time (p < 0.001) and session (p = 0.002), specifically, with each minute of classical music played, there was a 0.3 % reduction in CBFV (95 % confidence interval (CI): 0.2–0.4 %). Heart rate (HR) and respiratory rate (RR) decreased by 0.1 % (95 % CI: −0.2–0.0 %; p = 0.043) 0.3 % (95 % CI: −0.6 % to −0.1 %; p = 0.001), respectively, per minute of exposure. Each additional session resulted in a reduction of HR by 4.3 % and RR by 22.3 % from the baseline at the start of the intervention to minute 25 (both p < 0.001).

**Conclusions:**

Our pilot study shows only a very small effect of classical music such as Mozart's Symphony No. 40 in G *minor* in patients with SAH.

## Highlights

• Classical music can be used in the intensive care treatment of SAH patients.
• Classical Music has small effects on various physiological variables in SAH patients.
• Future studies should evaluate whether the results of this pilot study.
• Future studies evaluate whether a different selection of music might have a greater effect.

## Background

Aneurysmal subarachnoid hemorrhage (SAH) represents a neurological emergency and requires specialized management on a neurological intensive care unit (ICU) (Suarez et al., 2006, de Oliveira Manoel et al., 2016, Chung et al., 2021, Claassen and Park, 2022). Despite advances in diagnosis and treatment, it is estimated that one third of affected patients dies within the first 30 days after ictus, and another third requires lifelong support (Claassen and Park, 2022, Petridis et al., 2017). Cerebral vasospasm and the occurrence of delayed cerebral ischemia (DCI) are major contributors to this overall poor outcome (Claassen and Park, 2022, Vergouwen et al., 2010, Macdonald et al., 2014). One strategy to detect vasospasm besides clinical examinations and angiography examinations is to quantify the cerebral blood flow via transcranial doppler ultrasound (TCD) over the middle cerebral artery (MCA) (Macdonald et al., 2014, Kumar et al., 2016). Standard of care in patients with SAH is the calcium channel antagonist nimodipine, which has been shown to reduce the incidence of poor outcome while, however, not decreasing the occurrence of DCI or vasospasm (Allen et al., 1983, Dorhout Mees et al., 2007). Recently, a number of studies have investigated the effect of neuromodulation in patients with SAH, including invasive as well as non-invasive techniques such as facial nerve stimulation or transcutaneous electrical neurostimulation (Powell et al., 2022). Many of these approaches have demonstrated beneficial effects on reducing the occurrence of vasospasm or improving cerebral blood flow (Powell et al., 2022). Music exposition also represents a noninvasive stimulation technique with no known harmful side effects (Sihvonen et al., 2017). Previous studies on healthy participants have already confirmed positive physiological effects ranging from decreased blood pressure or heart rate to reduced release of stress associated hormones as well as decreased velocity of the cerebral blood flow (Bernardi et al., 2009, Trappe and Voit, 2016, Hou et al., 2017, Mir et al., 2021). Interestingly, it is classical music in particular – and especially the music by Wolfgang Amadeus Mozart – that is capable of generating such effects, which even led to the labelling of this phenomenon as “Mozart Effect” (Hughes, 2001).

Aim of this prospective pilot-study was to investigate the effect of classical music, specifically the *Symphony No 40 in g minor (KV 550)* by W. A. Mozart, on 1) cerebral blood flow velocity (CBFV) over the MCA, and 2) other physiological parameters (i.e., heart rate (HR), intracranial pressure (ICP)) in patients with SAH.

## Methods

### Study design and ethics

Patients suffering from SAH were enrolled in this prospective exploratory pilot-study. Written informed consent was obtained for all participants either from the patients directly or the legal proxy. The study was conducted according to the Declaration of Helsinki and was approved by the local ethical committee (Ethik-Kommission der Ärztekammer Hamburg No. PV7166). The protocol was registered with the German Clinical Trials Register (registration no. and search term: DRKS00022431, www.drks.de).

### Participants

Inclusion criteria were defined as 1) minimum of 18 years of age; 2) detection of an aneurysmal SAH using computed tomography (CT), magnetic resonance imaging (MRI), or lumbar puncture and an imaging procedure to detect the aneurysm, i.e., digital subtraction angiography (DSA), CT, or MRI angiography; 3) securing of the aneurysm using endovascular or microsurgical procedures. Exclusion criteria were 1) existing surgical wounds or drainages that were not compatible with the use of headphones or the TCD probe; 2) known hyp- or anacusis; 3) known pregnancy; 4) participation in an intervention study within the last 30 days, and 5) any other reasons that did not allow the participation.

### Experimental intervention

Our intervention was auditory exposure to *Symphony No 40 g minor (KV 550; Molto allegro; Andante; Menuetto. Allegretto and Allegro assai)* by W.A. Mozart (English Chamber Orchestra conductor: Jeffrey Tate) over approx. 25 min via headphones (model HDA 300, Sennheiser GmbH & Co. KG, Wedemark-Wennebostel, Germany), which are approved for medical-auditory purposes. The intervention was performed up to three times during the clinical course, with the first measurement no later than day 8 after SAH ictus. The study was conducted in a regular ICU setting. Continuous TCD measurements of the MCA were performed using a Multi-Dop® T Doppler system with 2mHz probe (Compumedics, Singen, Germany), which was mounted using a DiaMon® probe fixation holder (Compumedics, Singen Germany). All examinations were carried out between 10:00 a.m. and 2 p.m. by NE and PC.

Awake patients were able to freely choose the volume of the music; for all other patients, a volume of 70db was specified.

### Outcome measures

Primary outcome measure was the change in CBFV obtained by TCD in the MCA during the intervention. Furthermore, cortisol and arterial blood gas values were analyzed on all patients with invasive arterial blood pressure measurement or a central venous line immediately before and after the intervention.

Patients‘ vital signs monitoring included heart rate via a 3-lead electrocardiography, pulse oximetry including respiratory rate (RR) and invasive arterial blood pressure (Delta XL vital signs monitor, Drägerwerk, Lübeck, Germany).

ICP was only measured when an intraparenchymal ICP probe was in place or an external ventricular drainage (EVD) was present and clamped with a pressure transducer during the entire measurement period. The nasion with an upper body elevation of 30° was used as the zero-reference point for the foramen of Monro. The continuously measured TCD values were averaged to an average TCD value per minute; for all other vital parameters, a value was documented at the beginning of each full minute. All data w ere continuously collected and documented during the measurements.

Vasospasm was suspected by TCD if the mean flow velocity in the MCA was above 140 cm/s and above 120 cm/s in the ACA, respectively*.*

Patient demographics and SAH-specific data were obtained from the patient records. These data included demographic information, aneurysm size and location in relation to the anterior or posterior circulation and distinct clinical evaluation scores (e.g., World Federation of Neurosurgical Societies (WFNS) grading system, Fisher scale) as described before (Göttsche et al., 2023). The anterior circulation group contained aneurysms of the anterior cerebral, anterior communicating, internal carotid and middle cerebral artery. Posterior aneurysms included aneurysm of the basilar artery, posterior communicating artery, vertebral artery and its branches also as described before (Czorlich et al., 2015).

### Data pre-processing

Prior to conducting the statistical analysis, each dependent variable's values were normalized by dividing them by the initial recorded value from the respective session for that specific parameter. This normalization process ensures that the first value of any dependent variable for each session and individual is 1, thereby adjusting for unique baseline variations among individuals. Following this, any values falling outside the 1.5 interquartile range (IQR) were excluded from the analysis.

### Statistics

To account for the hierarchical structure of the data and the repeated measurements on the same participants, we employed a mixed-effects linear regression model. The fixed effects in our model included time, session (first vs. second vs. third session), their interaction (time * session), the number of days since ictus, age, sex, and the Fisher scale. The model also incorporated a random intercept for each participant to capture the variability in baseline values across different participants.

We used the statsmodels library (v0.13.2, statsmodels.org) with Python (v3.9.12, python.org) to fit this model. Specifically, the mixedlm function from the statsmodels.formula.api module was employed.

The model was fitted using the Restricted Maximum Likelihood (REML) approach, a common method for estimating variance components in mixed models, as it offers unbiased estimators of the variance components.

The results of this model provided insights into the fixed effects of each independent variable on the dependent measure, as well as the variability across participants.

Graphs were generated using matplotlib (v3.5.1) and seaborn (v0.11.2) with Python (v3.9.12, python.org).

### Sample size calculation

Based on the findings of Bernadi et al., we calculated that a sample size 20 patients was required to detect an α of 0.05 with a power of 0.8 (Bernardi et al., 2009).

## Results

### Study participants and demographics

In total 20 patients with SAH (14 women/ six men) with a mean of 52.3 ± 5.9 years of age were enrolled. Out of the 20 participants, 15 (75 %) received three sessions whereas five participants (25 %) only received two sessions due to discharge from hospital. The average interval between bleeding and first intervention session was 6.65 days (Standard Deviation (SD) = 3.25). The average interval between session one and session two was 3.55 days (SD=2.01), the average interval between session two and three 4.13 days (SD=1.55). Median WFNS scale was 1 (IQR: 1.0; 3.0) and median Fisher scale was 3 (IQR: 2.0; 4.0). Twelve patients (60 %) suffered from acute hydrocephalus requiring an external cerebrospinal fluid drainage. 8 Patients (40 %) developed a cerebral vasospasm during the clinical course. Demographic details of the study population are presented in Table 1.

**Table 1:** Baseline characteristics by study cohort.

**Variables**	**N = 20**
Age, years	52.3 ± 5.9
Sex (women), n (%)	14 (70)
WFNS	1 [1.0–3.0]
Fisher scale	3.0 [2.0–4.0]
Acute hydrocephalus, n (%)	12 (60)
Diameter of aneurysm, mm	5.8 ± 2.7
Number of aneurysms	1.2 ± 0.5
• Anterior circulation, n (%)	16 (80)
• Posterior circulation, n (%)	4 (20)
Vasospasm	8 (40)
Ventriculitis/Meningitis, n (%)	3(15)
VP Shunt, n (%)	4 (20)
Arterial Hypertension, n (%)	7 (35)
Smoking, n (%)	6 (30)
Length of stay, days	27.0 ± 9.9

Continuous variables are summarized with mean and standard deviation.

Abbreviations: WFNS = World Federation of Neurosurgical Societies Scale, mmHg = millimeter mercury, cm = centimeter

#### CBFV

**Fig. 1:**
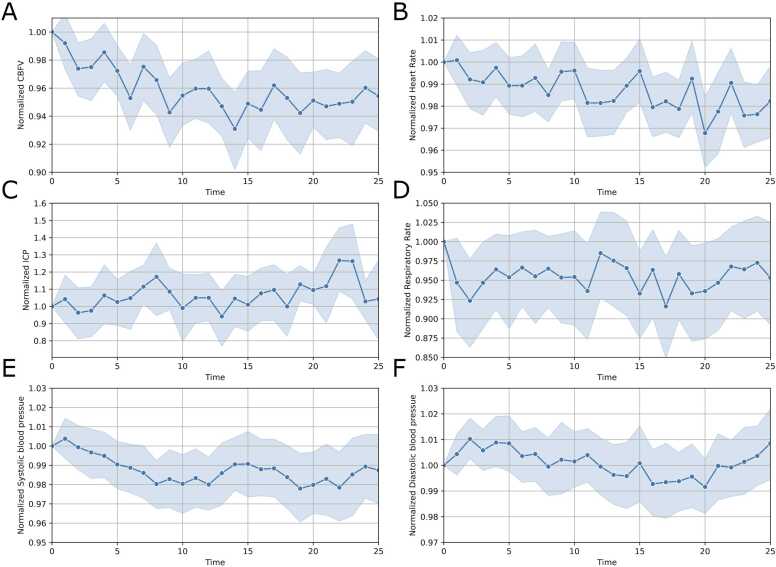
Change of continuously monitored parameters over time. Fig. 1 displays the change of continuously monitored parameters over the intervention time summarized over all patients and sessions (n = 55) period for A) the normalized CBFV (n = 54), B) the normalized heart rate (n = 53), C) the normalized ICP values (n = 9), D) the normalized respiratory rate (n = 28), E) the normalized systolic blood pressure values (n = 54) and F) the normalized diastolic blood pressure values (n = 54). The blue line represents the average of the value, while the shaded area indicates the standard deviation. CBFV = Cerebral Blood Flow Velocity; ICP = Intracranial pressure.

The mixed-effects linear regression model revealed significant associations with both time (p < 0.001) and session (p = 0.002), while no other variables demonstrated a significant impact. Specifically, with each minute of classical music played, there was a 0.3 % reduction in CBFV (95 % CI: 0.2–0.4 %, Fig. 1A). Notably, the number of sessions (repeated measures over different days) was correlated with a decrease in CBFV, resulting in a 2.6 % less reduction for each measurement compared to the previous intervention. Importantly, the interaction term between time and session was not statistically significant (p = 0.136), suggesting that the effects of time and session on CBFV are independent of each other. In contrast, the time period ictus-to-measurement showed no significant association (p = 0.113), suggesting that indeed rather the repeated music exposure and not merely the later timepoint after SAH onset affected CBFV. Therefore, this can be interpreted as follows: after 20 min of music played in session 1, there would be an overall reduction of 6 % in CBFV compared to the baseline and a 3.4 % reduction in session 2, independent of the time period of ictus-to-measurement.

### Heart rate

The mixed-effects regression model for HR highlighted notable associations with time, number of sessions, and days since the initial event. HR decreased by 0.1 % per minute of exposure (95 % CI: −0.2–0.0 %; p = 0.043, Fig. 1B). Each additional session resulted in a reduction of HR by 4.3 % from the baseline at start of the intervention to minute 25 (95 % CI: −5.5 % to −3.2 %; p < 0.001). Interestingly, length of stay showed the opposite effect with a 1.0 % increase in HR for every day after SAH onset (95 % CI: 0.7–1.2 %; p = 0.001). Other fixed effect factors demonstrated no significant influence on HR.

### Intracranial pressure

The mixed-effects regression model for ICP revealed significant associations related to sex, age, and the Fisher scale. Female sex was associated with a 11.4 % increase in ICP (95 % CI: 4.4–18.4 %; p = 0.002, Fig. 1C) over intervention from minute 0 to minute 25. Each increase in age by one year was associated with an ICP increase of 2.1 % as response to the intervention (95 % CI: 1.4–2.8; p < 0.001). Similarly, the Fisher scale displayed a significant positive relationship to the intervention, contributing to a 12.7 % increase in ICP (95 % CI: 5.4–19.9 %; p = 0.001). In contrast to CBFV and HR, the elapsed time, number of sessions, interaction between time and session, and the days since the initial event did not show significant impacts on ICP due to the small number of patients with multiple sessions with ICP measurement

### Respiratory rate

The mixed-effects regression model for RR uncovered significant relationships with time, the number of sessions, their interaction, and the days since the initial event. With every minute of the intervention RR was reduced by 0.3 % (95 % CI: −0.6 % to −0.1 %; p = 0.001, Fig. 1D). Each added session resulted in a substantial decrease of RR by 22.3 % from the baseline at the start of the intervention to minute 25 (95 % CI: −27.8to −16.7; p < 0.001). Notably, the interaction between time and session caused a rise in RR, with an increase of 0.2 % (95 % CI: 0.0–0.3 %; p = 0.03). This suggests that the combination of more time passing and more sessions had a measurable impact on making the RR slightly higher. Concerning the time since ictus, there was a significant increase in RR of 6.8 % for every day (95 % CI: 5.1–8.4 %; p = 0.008). Other investigated fixed effect factors demonstrated no significant effect on RR.

### Systolic blood pressure

The mixed-effects regression model for systolic blood pressure (SBP) highlighted significant findings with respect to time (p = 0.008) and days since the onset of the condition (p = 0.046), while the effects of session (p = 0.672) and the interaction between time and session (p = 0.071) were not statistically significant. Specifically, the model revealed that each minute of intervention was associated with a 0.1 % decrease in SBP (95 % CI: −0.2 % to −0.0 %). The effect of the number of sessions attended on SBP was not significant, indicating no clear trend of SBP changes with successive sessions. The interaction term between time and session, although close to significance, suggests a potential, yet non-conclusive, modification in the time effect on SBP by session, with a 0.0 % change per session (95 % CI: −0.0–0.1 %).

### Diastolic blood pressure

The mixed-effects regression model indicated significant associations with time (p < 0.001), session (p = 0.044), and the interaction between time and session (p = 0.001), alongside a significant effect of days since the onset of the condition (p = 0.001). Specifically, the model demonstrated that for each minute of intervention, there was a 0.1 % decrease in diastolic blood pressure (DBP) (95 % CI: −0.2 % to −0.1 %). Moreover, the influence of session was positive, indicating a 0.9 % increase in DBP for each additional session (95 % CI: 0.0–1.8 %). The interaction term suggests that the effect of time on DBP changes across sessions, with a 0.1 % increase in the rate of DBP reduction per session (95 % CI: 0.0–0.1 %).

All values of the first and last recorded timepoints, number of available timepoints and available sessions are presented in Table 2.

**Table 2:** Overview and Results of Investigated Physiological Parameters.

Variable	At First Recorded Timepoint	At Last Recorded Timepoint	Available Timepoints	Available Sessions
CBFV (cm/s)	75.6 95 % CI (66.9–84.4)	72.1 95 % CI (63.0–81.1)	4917	54
ICP (mmHg)	7.1 95 % CI (3.4–10.8)	7.9 95 % CI (3.8–12.0)	233	9
Heart Rate (beats/min)	70.1 95 % CI (66.5–73.6)	68.0 95 % CI (64.9–71.1)	1341	53
Respiratory Rate (breaths/min)	19.2 95 % CI (17.7–20.8)	17.6 95 % CI (15.9–19.2)	691	28
Diastolic Blood Pressure (mmHg)	71.0 95 % CI (68.0–74.0)	71.2 95 % CI (68.2–74.2)	1367	54
Systolic Blood Pressure (mmHg)	140.4 95 % CI (134.8–146.0)	138.4 95 % CI (133.1–143.8)	1367	54

CBFV = cerebral blood flow velocity; CI = Confidence Interval; ICP = intracranial pressure

Distribution of ICP measurement = 1 patient with 3 sessions and ICP measurement, 1 patient with 2 sessions and ICP measurement and 4 patients with ICP measurement during the first session

### Cortisol and arterial blood gases

Cortisol and arterial blood gas values were available in 53 measurements. There was no statistically significant difference between pre- and post-intervention values for pO_2_, pCO_2_ and pH (see Table 3) as well as for serum cortisol (154.0 µg/l ± 121.7 µg/l vs. 139.6 µg/l ± 122.1 µg/l, p > 0.05).

**Table 3:** Arterial Blood Gas Results.

Blood Gas Analysis n = 53	Pre-intervention	Post-intervention	p value
pO_2_ mmHg	79.7 ± 10.5	79.8 ± 12.9	> 0.05
pCO_2_ mmHg	34.0 ± 3.8	34.2 ± 3.8	> 0.05
pH	7.46 ± 0.02	7.47 ± 0.03	> 0.05
Cortisol μg/l	154.0 ± 121.7	139.6 ± 122.1	> 0.05

pCO_2_ = partial pressure of carbon dioxide; pO_2_ = partial pressure of oxygen

## Discussion

This pilot study examined the effects of classical music exposure in patients suffering from SAH. For this purpose, a number of physiological variables were measured and analyzed, including the CBFV over the MCA, ICP, HR, RR, and cortisol levels. Mozart’s *Symphony No. 40 in G minor* was chosen because of the studies that have already confirmed positive effects on certain physiological variables including a decreased CBFV (Bernardi et al., 2009, Trappe and Voit, 2016). Evolving from this knowledge gained in healthy participants, the present study focuses on the influence of classical music on patients with SAH.

We detected a decrease in CBFV under the influence of Mozart’s *Symphony No. 40 in G minor* with an increasing effect size if the intervention was repeated in additional sessions. Our findings in patients with SAH– despite potential disturbances in the cerebral vasculature – are in accordance with previous studies in healthy participants, which similarly reported a decrease in CBFV under the influence of classical music (Bernardi et al., 2009). The "Mozart Effect" was first described by Rauscher et al., who found an increased neurocognitive performance in healthy individuals after listening to Mozart’s *Sonata for two Pianos in D major K448* (Rauscher et al., 1993). The phenomenon of the Mozart Effect has been replicated in neurological patients, which included elderly patients with mild cognitive impairment and improved cognitive functions after listening to Mozart. A study conducted by Ren et al. reported changes in brain physiology associated with a higher degree of calmness and relaxation in preterm infants (Ren et al., 2019). Even though the “Mozart effect” has also been confirmed in animal models in terms of improved maze learning abilities in mice, the existence of the effect has been repeatedly a subject of discussion (Aoun et al., 2005, Verrusio et al., 2015). Today, a general consensus is that it is not Mozart per se but rather certain characteristics of his music that are causal to the observed effects, and which include a high degree of long-term periodicity, the absence of sung words, a skillful composition, or a catchy melody line (Trappe and Voit, 2016, Verrusio et al., 2015). The underlying neurobiological and psychological mechanisms of music-based interventions involve numerous neural systems such as the system for reward, affect regulation, or activity-driven plasticity of the brain (Sihvonen et al., 2017). A growing body of evidence supports the assumption that noninvasive brain stimulation with music represents a therapeutic tool, especially in the context of neurological diseases such as stroke, movement disorders, epilepsy or mild cognitive impairment (Sihvonen et al., 2017, Cacciafesta et al., 2010). This is exemplified in a higher neurocognitive capacity in patients with major MCA stroke or a decline in seizures in epilepsy patients in the context of music interventions (Särkämö et al., 2008, Quon et al., 2021, Bhandarkar et al., 2024, Toader et al., 2023). In line with such findings, we demonstrate that musical intervention is feasible in neuro-ICU patients, and can trigger a physiological response. Repeated interventions might lead to stronger effects, hence suggesting that a study protocol with a higher intensity in terms of exposure time might result in a more robust and amplified response, and should be considered in future investigations.

Regarding the ICP, the results revealed associations related to sex, age, and the Fisher scale. Specifically, being female was associated with an increase in ICP over the course of the intervention, and every additional year of age correlated with a rise in ICP. The finding that being female was associated with an increase in ICP is in contrast to previous published data that women tend to respond better regarding cardiovascular effects (Koenig and Thayer, 2016). The Fisher scale also displayed a significant positive relationship. In contrast, the elapsed time, number of sessions, interaction between time and session, and the days since the initial event did not show significant impacts on ICP due to the small number of patients with multiple sessions and ICP measurement. Furthermore, there were no relevant changes in the blood gas analyzes like a hypercapnia resulting in an increase of the ICP. All measured ICP values (Table 2) were within the normal range and therefore have no clinical relevance despite the statistical significance.

The analyses for HR as well as for RR support notable associations with time, number of sessions, and days since the initial event. Both show a decreasing trend over the initial phase that reverses as the days since the initial event progress. The fact that both tend to respond in a similar way may reflect the synchronicity of both the cardiovascular and the respiratory system.

Recent evidence suggests that the physiological effects of classical music may vary depending on the patient's state of consciousness. While music has been shown to reduce stress markers such as cortisol and lower sedative requirements in awake patients undergoing regional anesthesia, studies in sedated or unconscious patients (e.g., in ICU settings or under general anesthesia) indicate more variable responses. Some findings report enhanced sedation or reduced anesthetic requirements, while others show limited changes in vital parameters. These observations highlight the complexity of isolating music-induced effects and underscore the need for further research to better understand the influence of auditory stimulation across different levels of consciousness (Kühlmann et al., 2018, Fu et al., 2019, Fu et al., 2021, Koelsch et al., 2011, Trappe, 2012).

Due to the heterogeneity of medications within the study cohort and the limited number of interventions performed in patients with impaired consciousness or under analgesia, we were unfortunately unable to investigate the potential influence of classical music on medication dosage, nor to assess whether the effects of music differ across varying levels of consciousness. We acknowledge this as one of the main limitations of our study.

Furthermore, the study is subject to additional important limitations that should be taken into consideration which are partially associated with the nature of a pilot study. Due to the absence of control groups, the specific effect of the chosen music (versus, e.g., other composers, genres, or absence of music) cannot be determined with certainty, and biasing effects of the study participation itself and presence of the investigator at the bed site cannot be ruled out. The absence of a control group is based on the ethical concept that a pilot study should first explore whether an effect can be expected in a new, previously unstudied population. The confounding factor of the chosen musical style appears to be negligible, as it has been shown that Mozart’s *Symphony No. 40 in G minor* has the greatest effect on physiological parameters, regardless of the personal musical taste, and furthermore, certain musical styles can lead to an opposing influence on vital parameters (Trappe and Voit, 2016, Bernardi et al., 2006). Furthermore, the intervention took place under regular ICU conditions including alarms, the presence of ICU staff, etc., which could interfere with the study intervention and was naturally not consistent between participants. However, the real-life setting represents also a strength of the study because it proves a physiological effect in a clinical environment. In summary, the aim of this study was to evaluate whether there is a fundamental effect of classical music on CBFV in patients with SAH, on which the sample size planning was based rather than demonstrating a relevant effect of the intervention on cerebral vasospasm or outcome.

## Conclusions

Our pilot study shows only a very limited clinical effect of classical music such as Mozart's Symphony No. 40 in G *minor* in patients with SAH despite the statistical significance. Future studies should aim to identify specific patterns in music that have beneficial effects on CBFV and, with equal importance, patterns that may be detrimental. This knowledge could be useful for modifying the patient environment in the ICU.

## Grant support

The study was supported by Compumedics Germany GmbH, Singen, Germany. Compumedics Germany GmbH had no influence on the study design, conduct of the investigations, evaluation and interpretation of the data.

## Ethical standards

This study was conducted according to the Declaration of Helsinki and was approved by the local ethical committee (Ethik-Kommission der Ärztekammer Hamburg No. PV7166). Written informed consent was obtained by every patient or legal proxy. The protocol of this prospective study was registered in the German Clinical Trials Register (registration no. DRKS 00022431. Registererd July 9th 2020 https://drks.de/search/de/trial/DRKS00022431)

## Conﬂict statement

1. Patrick Czorlich received consulting fees from Acasti Pharma no related to this study. All other authors declare that they have no conflicts of interest related to the submitted work.

2. **Grant support:** The study was supported by Compumedics Germany GmbH, Singen, Germany. Compumedics Germany GmbH had no influence on the study design, conduct of the investigations, evaluation and interpretation of the data.

## CRediT authorship contribution statement

**Nicolas Eden:** Writing – original draft, Investigation, Formal analysis, Data curation, Conceptualization. **Patrick Czorlich:** Writing – original draft, Resources, Methodology, Investigation, Funding acquisition, Formal analysis, Data curation, Conceptualization. **Jens Gempt:** Writing – review & editing, Resources. **Nils Schweingruber:** Writing – review & editing, Resources, Formal analysis, Data curation. **Marlene Fischer:** Writing – review & editing. **Jörn Grensemann:** Writing – review & editing. **Jennifer Sauvigny:** Writing – review & editing, Investigation. **Jan Bremer:** Writing – review & editing, Visualization, Software, Formal analysis, Data curation. **Marius Marc-Daniel Mader:** Writing – review & editing, Methodology, Formal analysis, Data curation, Conceptualization.

## Declaration of Competing Interest

The authors declare the following financial interests/personal relationships which may be considered as potential competing interests:
